# The incidence of feline injection site sarcomas in the United Kingdom

**DOI:** 10.1186/1746-6148-9-17

**Published:** 2013-01-22

**Authors:** Rachel S Dean, Dirk U Pfeiffer, Vicki J Adams

**Affiliations:** 1 School of Veterinary Medicine and Science, University of Nottingham, Sutton Bonington Campus, Loughborough, LE12 5RD, UK; 2 Veterinary Epidemiology & Public Health Group, Royal Veterinary College, Hawkshead Lane, Hatfield, Hertfordshire, AL9 7TA, UK; 3 Veterinary Epidemiology Consulting, Bury-St. Edmunds, PO Box 80, Suffolk, IP28 9BF, UK

**Keywords:** Feline injection site sarcomas, Incidence, Risk

## Abstract

**Background:**

Feline injection site sarcomas (FISS) are aggressive neoplasms that have been associated with vaccination. In North America the incidence estimates have varied from 1 case of FISS per 1,000-10,000 cats vaccinated. The aim of this study was to estimate the incidence of FISS in the United Kingdom (UK) in 2007. The ratio of FISS to vaccines sold in the UK was also estimated.

Fourteen FISS were diagnosed by a convenience sample of 34 small animal veterinary practices in the United Kingdom in 2007 and were used as the numerator for the incidence estimates. Denominator data was obtained from the computer systems of each practice. Considering that a single cause relationship with vaccination is not proven, three different denominators (number of cats registered, the number of cat consultations undertaken and the number of vaccination visits for cats at the practices) were used to express the potential variation in risk.

**Results:**

The incidence risk of FISS per year was estimated to be 1/16,000 -50,000 cats registered by practices, 1/10,000-20,000 cat consultations and 1/5,000-12,500 vaccination visits.

**Conclusion:**

When interpreting these findings, it needs to be taken into consideration that this sample of practices and their cats may not be representative of veterinary practices and cats at risk of FISS in the UK. However it can still be concluded with reasonable certainty that the incidence of FISS in the UK is very low.

## Background

It has been suggested that feline injection site sarcomas (FISS) are a potential serious adverse event to vaccination in cats [1]. The cause of FISS remains unknown, though various hypotheses regarding causation have been suggested e.g. vaccination, injection, trauma [2-5]. Therefore the population at risk of developing FISS (denominator) remains ill-defined though may be all cats, only cats visiting veterinary practices, only cats receiving injections/vaccinations or another population of cats. This makes incidence estimates for this disease difficult.

To date all studies that have estimated incidence or prevalence of FISS have been in North America. The most recent study, undertaken in 1998–1999 estimated the incidence of FISS to be 0.63 sarcomas/10,000 cats vaccinated or 0.32 sarcomas/10,000 doses of all vaccines administered [6]. This estimate included a numerator of 2 cats with FISS and denominator data from 40 practices. Only cats that had been vaccinated were included in the population at risk. In 1996, Lester et al. reported an incidence rate of 1.3 FISS per 1,000 vaccinated cats from a single practice [7]. This study included 18 cases of FISS in the numerator but the details of the denominator are unclear. In 1993 Kass et al. reported a rate of 1.2 cases of FISS per 10,000 feline leukaemia virus (FeLV) vaccinations or 1.5 cases of FISS per 10,000 rabies vaccinations. These estimates were based on 29 practices vaccination records and tumour submissions to a histopathology service [2]. Coyne et al. estimated a prevalence of 2.1 cases/10,000 cat visits or 3.6 cases/10,000 cats. There were 158 tumours in the numerator and 235 practitioners provided denominator data [8].

The current reporting system in the UK for adverse events to veterinary products, including vaccination, is coordinated by the Veterinary Medicines Directorate (VMD). The VMD encourages veterinarians, owners and members of the public to report potential adverse events via the Suspected Adverse Reaction Surveillance Scheme (SARSS). In 2007, 857 reports were received by the VMD pertaining to cats, 59 of which were related to suspected injection site sarcomas [9]. To date there are no published estimates of the incidence of FISS in the United Kingdom (UK). The aim of this study was to estimate the incidence of FISS across a sample of practices in 2007. A second aim was to estimate the frequency of FISS in relation to vaccines sold in the UK in 2007.

## Methods

This study was part of a larger epidemiology project about FISS in the United Kingdom that included the development of a histopathology definition for FISS and a case–control study to identify risk factors for FISS (manuscripts in preparation).

### Numerator data

To be included in this study each tumour had to meet the inclusion criteria for the larger epidemiological study and be diagnosed in 2007. Each tumour was examined independently by two specialist veterinary pathologists and, to be included, had to have a minimum of 7 (out of a possible 10) features identified as part of the histopathology study. The 10 features included the presence of: aggregates of lymphocytes, infiltrative margins, intralesional necrosis, perilesional scarring,/inflammation, adjuvant-like material in macrophages, medium-high mitotic rate, giant cells and types of cellular differentiation (manuscript in preparation, Dean et al.). To be included in the estimate of incidence the FISS had to be diagnosed at the practices for which denominator information was available.

### Denominator data

Small animal and mixed practices in the United Kingdom that routinely submitted samples to 4 diagnostic histopathology laboratories (Abbey Veterinary Services, Newton Abbot: Animal Health Trust, Newmarket, University of Glasgow, IDEXX laboratories Ltd, Wetherby) were invited to participate in an epidemiological study concerning FISS. A letter was sent to all 2330 practices twice, 3 months apart asking them to participate in the study. All practices that responded to this letter and joined the study were called type A practices. If practices that routinely used these histopathology laboratories later submitted a sample from a cat that was diagnosed a possible case of FISS they were again invited to join the study at the time of diagnosis. Practices that joined as a result of diagnosing a FISS were called type B practices.

For this study of the incidence of FISS only practices that used management software systems produced by two specific companies, that had agreed to provide data for this study, were selected. Written permission was requested from each practice to allow the software companies to provide the denominator data. The denominator information for the practices was extracted by the employees on one software system for their member practices and by the author for the other.

For the practices that participated, denominator data was extracted from 1^st^ January to 31^st^ December 2007. Three different sets of denominator data were extracted:

Denominator 1. The total number of cats registered at the selected practices at the end of 2007.

Denominator 2. The total number of consultations/examinations, for which a code was in the system (e.g. primary consultation, repeat consultation etc.) recorded for cats by the selected practices during 2007.

Denominator 3. The total number of vaccinations visits for which there was a code in the system for vaccination visit (e.g. booster vaccination, primary vaccination courses etc.), recorded for cats by the selected practices during 2007.

For each of the 3 denominators, 2 different estimates of incidence were calculated. One set of estimates of incidence was generated using data from the type A practices and the cases they reported. A second estimate was made using data from both type A and type B practices and the cases they reported.

### Estimation of incidence

The incidence estimates were generated using the general formula of:

$$\begin{array}{l} \text{Incidence}\;\text{of}\;\text{FISS}\;\text{in}\;\text{the}\;\text{UK}\\ =\frac{\text{Number}\;\text{of}\;\text{new}\;\text{cases}\;\text{of}\;\text{FISS}\;\text{in}\;2007}{\text{Number}\;\text{of}\;\text{cats}\;\text{at}\;\text{risk}}\text{in}\;2007 \end{array}$$

### Ratio between FISS and number of vaccines sold

The total number of feline vaccines sold to practices in the UK in 2007 was provided by the pharmaceutical industry. The Royal College of Veterinary Surgeons register of practices was then used to identify how many registered practices did small animal work and therefore were likely to use feline vaccines. All 100% farm animal/equine practices and referral practices were removed from the list as they were unlikely to administer cat vaccines or diagnoses FISS. The number of FISS cases diagnosed in the sample of practices used in this study was used to calculate the number of FISS/practice in this study in 2007. This was then multiplied by the total number of practices that conduct small animal work in the UK to estimate how many FISS cases may have been diagnosed in the United Kingdom in 2007.

## Results

### Participating practices

**Figure 1:**
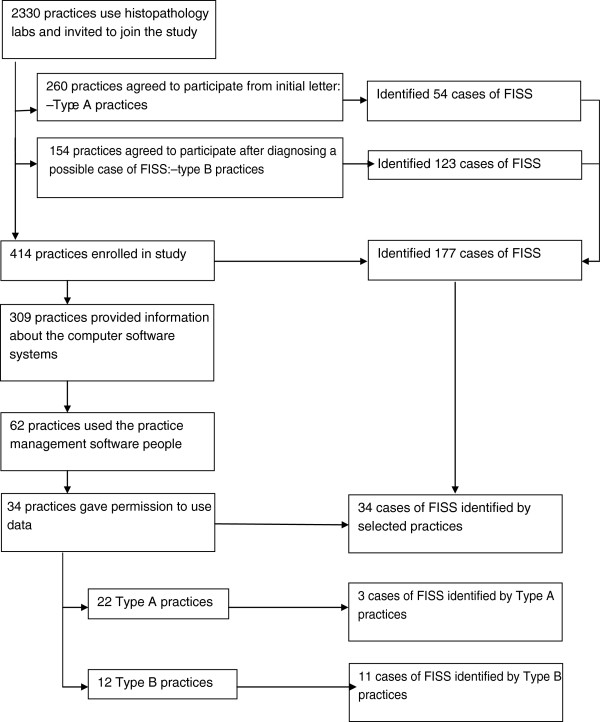
**Flow diagram demonstrating how practices and Feline injection site sarcoma (FISS) tumours were selected for inclusion in the incidence estimates.** Practice type A responded by initial letter of invitation at the start of the study; Practice type B responded to the pathology report addendum after identifying a case during the study period.

Of the 2330 practices that routinely submitted pathology samples to the 4 collaborating laboratories, 260 agreed to take part in the epidemiology study (response rate 11.2%.) These 260 practices diagnosed 54 FISS in 2007 that met the inclusion criteria for the study. A further 154 practices joined the study after identifying a FISS. These practices diagnosed 123 cases in 2007 that met the inclusion criteria for the study. A total of 177 cases of FISS were therefore identified by the enrolled practices in 2007.Of the enrolled 414 practice, 309 provided information about whether they were computerised for patient records and 94.5% were (n = 292). Sixty two practices used the two companies that had agreed to provide denominator data. Of these 34 practices gave permission to use the data for the estimation of incidence. This included 22 type A practices that had identified 3 FISS in 2007, and 12 type B practices that identified 11 FISS in 2007 (Figure 1).

### Estimates of incidence risk of FISS per year

The incidence estimates were very low for all types of denominators and numerators, ranging from 1 FISS per 50,000 cats registered to 1 FISS per 5,000 cat vaccination visits (Table 1).

**Table 1:** Estimates of incidence of FISS in the United Kingdom in 2007

**Number (type) of practices**	**Numerator (FISS)**	**Denominator**		**Incidence risk of FISS per year**	
22 (A only)	3	122,736	cats registered	0.00002 FISS per cat registered	1 FISS per 50,000 cats registered
34 (A and B)	14	206,942	cats registered	0.00007 FISS per cat registered	1 FISS per16,666 cats registered
22 (A only)	3	54,732	cat consultations	0.00005 FISS per cat consultations	1 FISS per 20,000 cat consultations
34 (A and B)	14	102,696	cat consultations	0.00001 FISS per cats consultations	1 FISS per 10,000 cat consultations
22 (A only)	3	38,205	cat vaccination visits	0.00008 FISS per cat vaccination visits	1 FISS per12,500 cat vaccination visits
34 (A and B)	14	58,516	cat vaccination visits	0.0002 FISS per cat vaccination visits	1 FISS per 5,000 cat vaccination visits

### Ratio between FISS and number of vaccines sold

From the RCVS register of practices it was estimated that there were 3399 practices that did small animal work (excluding referral practices) and were therefore likely to vaccinate cats. The pharmaceutical industry stated that 3,607,510 doses of feline vaccines were sold to practices in 2007.

Fifty four cases were identified from 260 type A practices in 2007, which is a rate of 0.21 FISS/practice per year. If these practices were representative of the 3399 practices that did small animal work in the UK in 2007, then 706 FISS may have occurred across all small animal practices in 2007. This would suggest that 0.0002 FISS occur per vaccine sold per year or 1 FISS occurs for every 5,000 vaccines sold per year.

The 414 type A and type B practices identified 177 FISS, which is a rate of 0.42 cases/practice per year. If these practices were representative of the 3399 practices that did small animal work in the UK in 2007, then 1453 FISS may have occurred in 2007. This would suggest that 0.0004 FISS per vaccine sold per year or 1 FISS occurs for every 2,500 vaccine sold per year.

## Discussion

The estimates of incidence of FISS in this study were very low for all denominators used and it therefore seems justified to conclude that the incidence risk of cats suffering from FISS is very low in United Kingdom. The incidence estimates reported in this study lie within the range of incidence estimates for North America previously reported in the veterinary literature [2,5,7].

It was not possible to calculate the true incidence risk of FISS in the UK as neither accurate numerators or denominators were available. Establishing accurate estimates is very difficult for rare diseases. This is compounded by the fact that the epidemiological understanding of the risk factors associated with FISS development remains poor, therefore the population at risk is unknown. It has been suggested that vaccination could be a risk factor for FISS, but the evidence for a causal relationship is weak, and therefore vaccinated cats are likely to be an inappropriate denominator [2,3]. It has also been suggested that other injectable products may play a role in FISS development, [4,5], hence multiple denominators were used. Whatever the causal factors for FISS, the risk of developing this tumour to an individual cat is very low.

In the current study numerator and denominator data were only available for a small number of practices. Incidence and prevalence estimates for FISS in previous studies have also been based on a small number of practices [2,6,7]. The reasons why some practices chose to be in this study, used particular computer software systems and allowed access the data, were no doubt numerous. This small sample of practices is unlikely to, but may, represent the general population of veterinary practices in the UK. It was not possible to include all practices in the UK in this study but more practices would have potentially generated more reliable estimates of FISS. It is unknown what an ‘average’ practice is like so the external validity of this study is highly questionable. The incidence estimates for type A practices alone were much lower than the incidence estimates when data from type A and type B practices were used. This is to be expected as type B practices joined the study because they had identified a possible FISS. It is unclear whether type A practices or type A and type B practices are more representative of small animal practices in the UK.

The estimates of incidence given in this study were based on a number of assumptions, as limited data were available. The numerator used in the final estimates was low due to the limited number of practices with denominator data available and the low frequency of FISS in the feline population. It is possible that more FISS did occur within these practices but were not reported to the investigator. Potential reasons not to report a FISS amongst others could be lack of permission from the owner to enter the tumour into the study, a lack of time to complete the paperwork or an owner declined histological examination of a FISS.

Multiple denominators were used in this study as it was not known what/who the population at risk was. It is possible that all cats are at risk of developing FISS, or just those visiting veterinary practices. Alternatively it may only be cats that were vaccinated/injected if indeed this is required for tumorigenesis. The denominators were chosen either because they were the same as those used in previous studies so comparisons could be made, were associated with some of the hypotheses surrounding FISS or it was possible to extract this information from both computer software systems with relative ease [2,6,7]. The number of cats currently registered should provide a reasonable estimate of the number of cats who could potentially be identified as cases if they developed FISS and probably represent the most reliable denominator. The number of cat consultations recorded by a practice was used as another denominator as this could be another possible population at risk. The total number of consultations was used regardless of whether it was an initial or repeat consultation since it was not possible to separate the types of consultations for all practices from the information provided. It is likely that some, but not all, cats that visit a veterinary practice would have received an injection such as a vaccination or other product. Therefore the first two denominators may be a more realistic representation of the population at risk, therefore these estimates may be the most accurate. It was not possible to determine from the practice data how many injections had been given to cats in 2007. This would provide very valuable data if it became available. The number of vaccinations recorded as being administered was used as the third denominator. However, this does not take account of the currently accepted theory that other vaccines may be involved in the aetiology of FISS, and it therefore may be the most inaccurate denominator to use, of the three presented here.

The reliability of the denominator data is questionable for many reasons. The data came from the two different sources and was extracted from practices in two different ways. The way in which cats are added and removed from a practice database is unknown and probably differs between practices. The accuracy of the extracted data was entirely reliant on the accuracy of the entered data. Practices may code vaccinations, consultations etc. in many different ways so a code in a software system in one practice may mean something else entirely by another practice. The denominators used in this study had many limitations and more sophisticated methods need to be explored to provide more accurate data about the vet visiting population of cats and disease prevalence and incidence. This issue is currently be addressed in the UK by the work of SAVSNET (http://www.liv.ac.uk/savsnet) and VetCompass (http://www.rvc.ac.uk/vetcompass) who are involved in developing methods for disease surveillance in small animal veterinary practices in the United Kingdom.

This study indicates that more injection site sarcomas occur in the United Kingdom than were reported to the Veterinary Medicines Directorate (VMD) in 2007 [9]. This study only included the practices enrolled in the study and 4 diagnostic histopathology laboratories. It is not possible to make estimates of how many more FISS may have been diagnosed at other laboratories in 2007 but there is no doubt that there would have been more FISS diagnosed during this period. If the caseload of these practices does represent all practices in the United Kingdom in, the number of cases of FISS could be over 1400 though the true number of FISS remains unknown. It is unknown whether or not the tumours in this study have also been reported to the VMD. Or if the cases reported to the VMD would fulfil the criteria used in this study. There are probably numerous reasons why the number of FISS reported to the VMD was lower than the number identified in this study. The VMD’s suspected adverse reaction surveillance scheme (SARSS) is passive whereas this study was a more active form of surveillance. The number of cats in the UK is thought to be around 10.3 million, so this disease is still very rare in the cat population in the UK [10].

Vaccination protocols vary between countries, so if vaccination is involved in the aetiology of FISS, then the incidence of this disease would be expected to be different. For example, rabies vaccination is not a routine procedure for cats in the UK but is in other parts of the world. It is interesting that the incidence estimates in this study are similar to, but not the same, as some of those from North America [2,6,8], yet vaccination protocols differ between the countries. This highlights the fact that it is not possible to extrapolate these estimates of incidence to other countries but it appears that these tumours are rare in all of the cat populations studied.

The ratio of FISS to the number of vaccines sold in the UK was estimated, as the number of vaccines sold has been used before in studies in North America. The number sold does not necessarily equate to number administered (as some batches maybe faulty or exceed their shelf-life) and if the enrolled practices are not representative of the veterinary practices in the UK then the estimate of the number of FISS in 2007 will be inaccurate. It is interesting to note that the estimated ratio of FISS per vaccines sold (1 FISS/2,500 - 5,000 vaccines sold), and the incidence estimate that used vaccinations visits recorded (1 FISS/5,000-12,500 vaccines recorded as administered) are different. One possible explanation is that when a cat receives two consecutive vaccinations (e.g. kitten and re-vaccination protocols) it is only recorded in the system once. These estimates of incidence are made in two completely different ways and using different numbers of cases and practices. It is therefore only possible to speculate about which the most accurate is.

## Conclusion

Many assumptions were made when calculating these estimates of incidence so they must be interpreted and reported with caution. However it can be concluded that whilst these tumours are more common than currently reported to the VMD, the incidence is still very low in the UK cat population. If more accurate estimates of the incidence of FISS are required further work is needed. However FISS is a rare disease and there are many more feline diseases that have much more significant morbidity and mortality and therefore a greater effect on cat welfare than this tumour.

## Competing interests

RD is currently the Director of the Centre for Evidence-based Veterinary Medicine, which is supported by an unrestricted grant by Novartis Animal Health and The University of Nottingham.

## Authors’ contributions

RD undertook this work as part of a PhD thesis at the Royal Veterinary College and Animal Health Trust. VJA and DP were involved in the design, conduct and reporting of this study. All authors read and approved the final manuscript.
